# Trends in Androgen Deprivation Use in Men With Intermediate-Risk Prostate Cancer Who Underwent Radiation Therapy

**DOI:** 10.1016/j.adro.2022.100904

**Published:** 2022-02-04

**Authors:** Vishesh Agrawal, Xiaoyue Ma, Jim C. Hu, Christopher E. Barbieri, Himanshu Nagar

**Affiliations:** a Department of Radiation Oncology, Weill Cornell Medicine, New York, New York; b Division of Biostatistics and Epidemiology, Department of Health Care Policy and Research, Weill Cornell Medicine, New York, New York; c Department of Urology, Weill Cornell Medicine, New York, New York

## Abstract

**Purpose:**

Until 2018, National Cancer Comprehensive Network guidelines recommended androgen deprivation therapy (ADT) for all men with intermediate-risk prostate cancer who had undergone radiation therapy. Intermediate risk was stratified as favorable and unfavorable in 2018, and ADT recommendation was limited to men with unfavorable intermediate-risk prostate cancer. Data suggesting this stratification and treatment deintensification were first published in December 2013. This study characterizes US national trends for demographic, clinical, and socioeconomic factors associated with ADT use in men with intermediate-risk prostate cancer who have undergone definitive radiation therapy.

**Methods and Materials:**

This retrospective cohort study examined 108,185 men in the National Cancer Database who were diagnosed with intermediate-risk prostate cancer from 2004 to 2016. Temporal trends in demographic, clinical, and socioeconomic factors among men with intermediate-risk prostate cancer and associations with the use of ADT were characterized.

**Results:**

In total, 108,185 men diagnosed with intermediate-risk prostate cancer underwent radiation therapy from 2004 to 2016. Of these men, 41.09% received ADT. Among the 60,705 men with favorable intermediate-risk prostate cancer, 32.06% received ADT. Among the 47,480 men with unfavorable intermediate-risk prostate cancer, 52.64% received ADT. On multivariate analysis, use of ADT was associated with age and year of diagnosis; being a race other than White; having government-based insurance; having a higher prostate-specific antigen level, tumor stage, and Gleason score; receiving treatment at a nonacademic center; and receiving external beam radiation therapy alone.

**Conclusions:**

The findings highlight that ADT use is variable in men undergoing definitive radiation therapy for intermediate-risk prostate cancer, with the data suggesting that several clinical and socioeconomic disparities influence its use. The findings suggest that a significant proportion of men with favorable intermediate-risk prostate cancer receive ADT and remain candidates for treatment de-escalation, whereas a significant proportion of men with unfavorable intermediate-risk prostate cancer may be undertreated when ADT is omitted.

## Introduction

Prostate cancer is a significant health burden, with 191,930 new cases estimated and 33,330 deaths in the United States in 2020.^1^ Until 2018, National Cancer Comprehensive Network guidelines recommended androgen deprivation therapy (ADT) for all men with intermediate-risk prostate cancer who had undergone radiation therapy. Intermediate risk was stratified as favorable and unfavorable in 2018, and ADT recommendation was limited to men with unfavorable intermediate-risk prostate cancer. Data suggesting this stratification and treatment de-intensification were initially published in December 2013.^2^ A recent secondary analysis of the Radiation Therapy Oncology Group (RTOG) 9408 randomized trial confirmed no improvement in the rate of distant metastasis or prostate cancer–specific mortality with the use of 4 months of ADT compared with radiation therapy alone for patients with favorable intermediate-risk prostate cancer.^3^ In contrast, the rate of distant metastasis or prostate cancer–specific mortality was reduced with ADT in patients with unfavorable intermediate-risk prostate cancer. The present study aimed to examine the use of ADT in men with intermediate-risk prostate cancer and characterize national trends.

## Methods

### Data source

The National Cancer Database (NCDB) is an oncology-focused national database established by the American College of Surgeons and the Commission on Cancer of the American College of Surgeons. The NCDB tabulates longitudinal data from more than 70% of all new cancer diagnoses annually, encompassing more than 1500 hospitals across all 50 US states. The collected data include cancer characteristics, primary and adjuvant management, and long-term outcomes, as well as patient demographic information such as age, sex, race, educational level, income, and insurance status. The present study followed the Strengthening the Reporting of Observational Studies in Epidemiology (STROBE) reporting guideline for cohort studies. Because the study used deidentified data from the NCDB database, the requirement for formal institutional review and the need for informed patient consent were waived, consistent with the policies of Weill Cornell Medicine.

### Study population

Men diagnosed with intermediate-risk prostate cancer who underwent radiation therapy from 2004 to 2016 were included in this study. Intermediate-risk prostate cancer was defined as either a Gleason score of 7, a prostate-specific antigen (PSA) level of 10 to 20 ng/mL, or a clinical stage of T2b-T2c. A subgroup analysis of men with favorable and unfavorable intermediate-risk prostate cancer was based on National Comprehensive Cancer Network criteria. Favorable intermediate risk was defined as the presence of 1 intermediate risk factor. Unfavorable intermediate risk was defined as a Gleason score of 4 + 3 = 7 or more than 1 intermediate risk factor. Intermediate risk factors were defined as a Gleason score of 7, a PSA level of 10 to 20 ng/mL, or a clinical stage of T2b-T2c. Men who had contraindications to or refused ADT were excluded from the analysis.

### Statistical analysis

Demographic characteristics and clinical characteristics were compared for all patients who were treated with ADT and who were not in bivariate analysis. The Pearson χ^2^ test was performed for categorical variables, with frequencies and percentages reported, and the Wilcoxon sum rank test was performed for continuous variables, with medians and interquartile ranges reported. The Cochran-Armitage test was used to identify significant trends in the use of ADT with time and by therapy treatment. Multivariate logistic regression analyses were used to examine demographic and clinical factors associated with ADT use, with adjusted odds ratios reported. All tests were 2-sided and were considered significant at an α level of .05. All analyses were performed using SAS software, version 9.4 (SAS Institute Inc).

## Results

**Fig. 1:**
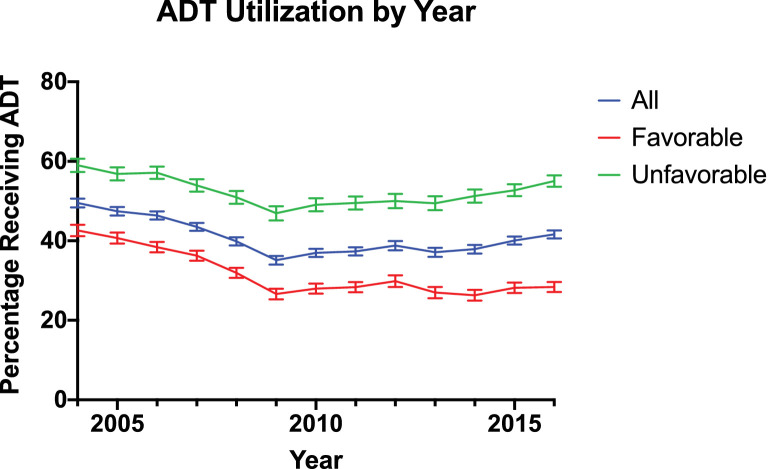
Trend in patients using androgen deprivation therapy as a percentage of all patients with intermediate-risk prostate cancer and subsets of patients with favorable intermediate-risk and unfavorable intermediate-risk prostate cancer from 2004 to 2016.

Demographic and clinical characteristics are presented in Table 1. In total, 108,185 men diagnosed with intermediate-risk prostate cancer underwent radiation therapy from 2004 to 2016. A flow diagram outlining the cohort selection is shown in Figure E1 in the Supplement. Of note, 5957 men with intermediate-risk prostate cancer who underwent radiation therapy who refused (4624) or had contraindications to (1333) ADT were excluded from the analysis. Of these men, 41.09% received ADT. The median ages of men who received and did not receive ADT were 69 and 68 years, respectively. Of patients with recorded treatment dates, ADT was initiated a median of 70 days before the start of radiation therapy, with an interquartile range of 107 days to 47 days before radiation therapy. White men composed 78.7% of the cohort, and Black men composed 17.2%. Approximately 85% of the cohort had a Charlson-Deyo Comorbidity Index score of 0. The median PSA level was 6.8 ng/mL. Among the 60,705 men with favorable intermediate-risk prostate cancer, ADT was used in 32.06%. Among the 47,480 men with unfavorable intermediate-risk prostate cancer, ADT was used in 52.64%. The trend of ADT use for the entire cohort and each subgroup from 2004 to 2016 is shown in Figure 1.

**Table 1:** Demographic and clinical characteristics of men with intermediate-risk prostate cancer from 2004 to 2014 who underwent definitive radiation therapy with or without ADT

Patient characteristic	All patients, No. (%) (N = 108,185)	No ADT, No. (%) (n = 63,731)	ADT, No. (%) (n = 44,454)	*P*
Year of diagnosis				
2004	8086 (7.47)	4081 (6.40)	4005 (9.01)	<.001
2005	8360 (7.73)	4393 (6.89)	3967 (8.92)	
2006	9156 (8.46)	4911 (7.71)	4245 (9.55)	
2007	9593 (8.87)	5418 (8.50)	4175 (9.39)	
2008	8916 (8.24)	5361 (8.41)	3555 (8.00)	
2009	7387 (6.83)	4792 (7.52)	2595 (5.84)	
2010	8377 (7.74)	5279 (8.28)	3098 (6.97)	
2011	8475 (7.83)	5311 (8.33)	3164 (7.12)	
2012	6767 (6.26)	4141 (6.50)	2626 (5.91)	
2013	7046 (6.51)	4430 (6.95)	2616 (5.88)	
2014	7647 (7.07)	4748 (7.45)	2899 (6.52)	
2015	8926 (8.25)	5350 (8.39)	3576 (8.04)	
2016	9449 (8.73)	5516 (8.66)	3933 (8.85)	
Age, y				
<60	15,366 (14.2)	10,093 (15.8)	5273 (11.9)	<.001
60 to <70	43,827 (40.5)	26,848 (42.1)	16,979 (38.2)	
70 to <80	43,424 (40.1)	23,870 (37.5)	19,554 (44.0)	
80 to 90	5568 (5.15)	2920 (4.58)	2648 (5.96)	
Gleason score				
3 + 3	17,074 (15.8)	11,565 (18.1)	5509 (12.4)	<.001
3 + 4	62,150 (57.4)	38,345 (60.2)	23,805 (53.5)	
4 + 3	28,961 (26.8)	13,821 (21.7)	15,140 (34.1)	
Median PSA level, ng/mL	6.80 (5.00-10.3)	6.40 (4.80-9.60)	7.40 (5.20-11.2)	<.001
PSA categories, ng/mL				
≤4	12,255 (11.3)	7736 (12.1)	4519 (10.2)	<.001
4 to ≤10	67,389 (62.3)	41,703 (65.4)	25,686 (57.8)	
10 to ≤20	28,541 (26.4)	14,292 (22.4)	14,249 (32.1)	
Clinical T stage				
T1a-T2a	87,566 (80.9)	53,329 (83.7)	34,237 (77.0)	<.001
T2b	9333 (8.63)	4746 (7.45)	4587 (10.3)	
T2c	11,286 (10.4)	5656 (8.87)	5630 (12.7)	
Charlson-Deyo Comorbidity Score				
0	92,082 (85.1)	54,298 (85.2)	37,784 (85.0)	.206
1	12,757 (11.8)	7521 (11.8)	5236 (11.8)	
2	2468 (2.28)	1406 (2.21)	1062 (2.39)	
≥3	878 (0.81)	506 (0.79)	372 (0.84)	
Race				
White	85,192 (78.7)	50,281 (78.9)	34911 (78.5)	.084
Black	18,591 (17.2)	10,925 (17.1)	7666 (17.2)	
Other	4402 (4.07)	2525 (3.96)	1877 (4.22)	
Primary payer				
Medicare, Medicaid, or other government insurance	69,052 (63.8)	39,013 (61.2)	30,039 (67.6)	<.001
Private	36,290 (33.5)	23,056 (36.2)	13,234 (29.8)	
Uninsured	1367 (1.26)	810 (1.27)	557 (1.25)	
Unknown	1476 (1.36)	852 (1.34)	624 (1.40)	
Median annual income, USD				
<38,000	20,453 (18.9)	11,964 (18.8)	8489 (19.1)	<.001
38,000-47,999	23,268 (21.5)	13,745 (21.6)	9523 (21.4)	
8,000-62,999	24,979 (23.1)	14,399 (22.6)	10,580 (23.8)	
≥63,000	39,485 (36.5)	23,623 (37.1)	15,862 (35.7)	
Education: High school diploma				
≥21%	21,359 (19.7)	12,293 (19.3)	9066 (20.4)	<.001
13%-20.9%	27,651 (25.6)	16,347 (25.6)	11,304 (25.4)	
7%-12.9%	31,318 (28.9)	18,307 (28.7)	13,011 (29.3)	
<7%	27,857 (25.7)	16,784 (26.3)	11,073 (24.9)	
Distance from treatment facility, miles				
≤60	100,349 (92.8)	58,343 (91.5)	42,006 (94.5)	<.001
60-120	3894 (3.60)	2527 (3.97)	1367 (3.08)	
>120	3942 (3.64)	2861 (4.49)	1081 (2.43)	
Location type				
Metropolitan	89,980 (83.2)	53,296 (83.6)	36,684 (82.5)	<.001
Urban	15,855 (14.7)	9167 (14.4)	6688 (15.0)	
Rural	2350 (2.17)	1268 (1.99)	1082 (2.43)	
Facility type				
Community	9819 (9.08)	4992 (7.83)	4827 (10.9)	<.001
Comprehensive	50,021 (46.2)	28,544 (44.8)	21,477 (48.3)	
Academic	34,198 (31.6)	21,678 (34.0)	12,520 (28.2)	
Integrated	14,147 (13.1)	8517 (13.4)	5630 (12.7)	
Facility location				
New England	6862 (6.34)	3369 (5.29)	3493 (7.86)	<.001
Mid-Atlantic	18,385 (17.0)	10,252 (16.1)	8133 (18.3)	
South Atlantic	27,332 (25.3)	17,004 (26.7)	10,328 (23.2)	
Central: East North	19,811 (18.3)	11,815 (18.5)	7996 (18.0)	
Central: East South	6735 (6.23)	4189 (6.57)	2546 (5.73)	
Central: West North	7491 (6.92)	3990 (6.26)	3501 (7.88)	
Central: West South	4466 (4.13)	2568 (4.03)	1898 (4.27)	
Mountain	3794 (3.51)	2321 (3.64)	1473 (3.31)	
Pacific	13,309 (12.3)	8223 (12.9)	5086 (11.4)	
Type of radiation therapy				
EBRT	70,407 (65.1)	38,202 (59.9)	32,205 (72.4)	<.001
BT	23,680 (21.9)	17,362 (27.2)	6318 (14.2)	
Combination of EBRT and BT	14,098 (13.0)	8167 (12.8)	5931 (13.3)	
Intermediate risk Stratification				
Favorable	60,705 (56.1)	41,244 (64.7)	19,461 (43.8)	<.001
Unfavorable	47,480 (43.9)	22,487 (35.3)	24,993 (56.2)	

*Abbreviations:* ADT = androgen deprivation therapy; BT = brachytherapy; EBRT = external beam radiation therapy.

**Fig. 2:**
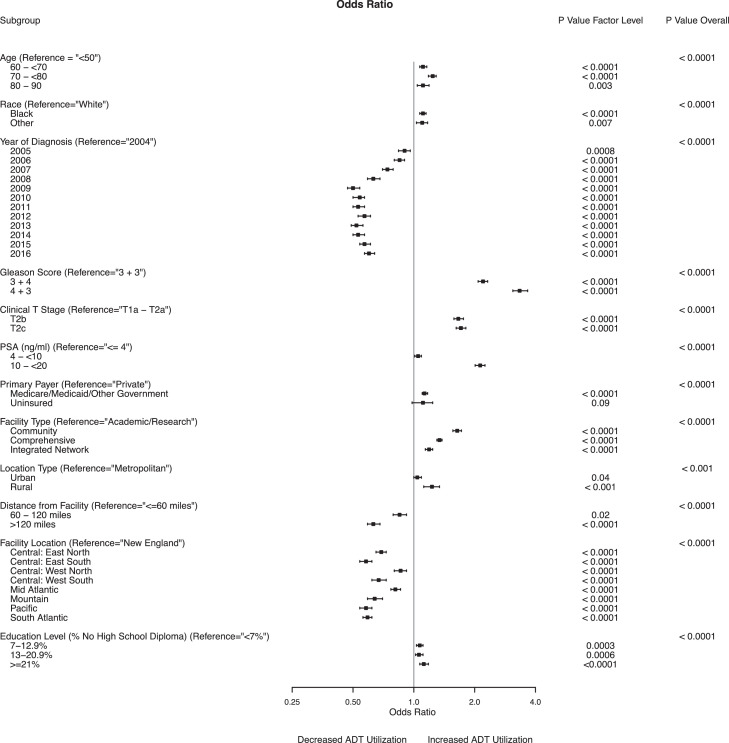
Multivariable logistic regression model estimating use of androgen deprivation therapy among patients with intermediate-risk prostate cancer.

Multivariable analyses for men with intermediate-risk prostate cancer who received ADT are presented in Figure 2 and in Table E1 in the Supplement. Men with PSA values greater than 10 ng/mL (odds ratio [OR], 2.13; 95% CI, 2.01-2.25; *P* <.001) and PSA values of 4 to 10 ng/mL (OR, 1.05; 95% CI, 1.01-1.09; *P* = .02) had an increased likelihood of receiving ADT compared with men with PSA values ≤4 ng/mL. Men with a Gleason score of 3 + 4 = 7 (OR, 2.20; 95% CI, 2.08-2.32; *P* < .0001) and a Gleason score of 4 + 3 = 7 (OR, 3.36; 95% CI, 3.09-3.66; *P* < .0001) were more likely to receive ADT than were men with a Gleason score of 3 + 3 = 6. A higher clinical tumor stage was associated with use of ADT (T2b: OR, 1.66; 95% CI, 1.58-1.76; *P* < .0001; T2c: OR, 1.71; 95% CI, 1.62-1.81; *P* < .0001, compared with T1a-T2a).

Black men had an increased likelihood of receiving ADT compared with White men (OR, 1.11; 95% CI, 1.07-1.15; *P* < .001). Older men had an increased likelihood of receiving ADT compared with men younger than 60 years (60 to <70 years: OR, 1.11; 95% CI, 1.07-1.16; *P* < .0001; 70 to <80 years: OR, 1.24; 95% CI, 1.18-1.29; *P* < .0001; 80-90 years: OR, 1.11; 95% CI, 1.04-1.19; *P* = .003).

Living more than 120 miles away from the treatment facility was associated with decreased ADT use compared with living less than 60 miles away (OR, 0.63; 95% CI, 0.59-0.68; *P* < .0001). Men whose treatment location was in the Mid-Atlantic (OR, 0.81; 95% CI, 0.77-0.86; *P* < .0001), Mountain (OR, 0.67; 95% CI, 0.59-0.70; *P* < .0001) or Pacific (OR, 0.58; 95% CI, 0.54-0.62; *P* < .0001) region were less likely to received ADT than were men with a New England facility location. Treatment in a community (OR, 1.64; 95% CI, 1.56-1.72; *P* < .001), comprehensive (OR, 1.34; 95% CI, 1.30-1.38; *P* < .0001), or integrated cancer center (OR, 1.19; 95% CI, 1.14-1.24; *P* < .0001) was associated with increased ADT use compared with treatment at an academic center. Men living in a rural area (OR, 1.23; 95% CI, 1.18-1.34; *P* < .0001) or an urban area (OR, 1.04; 95% CI, 1.00-1.09; *P* = .04) had an increased likelihood of receiving ADT compared with men living in a metropolitan area. Increased ADT use (OR, 1.12; 95% CI, 1.07-1.18; *P* < .001) was associated with a larger percentage of men without a high school diploma (>21% vs <7%).

**Fig. 3:**
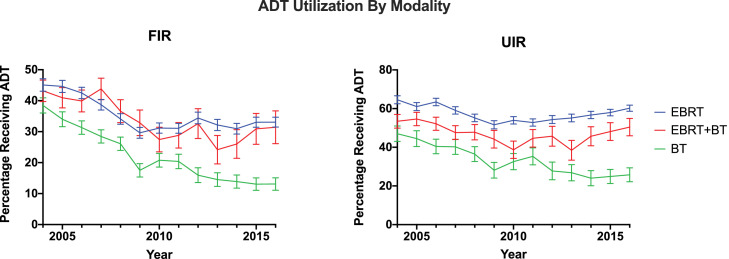
Trend in patients using androgen deprivation therapy as a percentage of patients receiving external beam radiation therapy (EBRT), EBRT with brachytherapy, or brachytherapy alone, grouped by favorable intermediate-risk and unfavorable intermediate-risk prostate cancer from 2004 to 2016.

Characteristics of ADT use by radiation therapy modality are shown in Table 1. Of 70,407 men treated with external beam radiation therapy (EBRT) alone, the percentage of men who received ADT decreased from 54.13% in 2004 to 47.18% in 2016 (*P* < .0001). Of 14,098 men treated with EBRT and brachytherapy (EBRT + BT), the percentage of men who received ADT decreased from 48.22% in 2004 to 43.13% in 2016 (*P* < .0001). Of 23,680 men treated with BT alone, the percentage of men who received ADT decreased from 40.9% to 17.42% during the study period (*P* < .0001) (Fig 3). This consisted of a decrease from 38.4% to 13.1% for patients with favorable intermediate-risk prostate cancer and a decrease from 47.0% to 25.7% for patients with unfavorable intermediate-risk prostate cancer.

## Discussion

Using an oncology focused national database cohort of men with newly diagnosed intermediate-risk prostate cancer who had undergone radiation therapy, we provide a descriptive analysis of the largest and most recent study to date, to our knowledge, exploring trends and factors associated with the use of ADT. Recent data have suggested that men with favorable intermediate-risk prostate cancer do not benefit from the addition of ADT, whereas men with unfavorable intermediate-risk prostate cancer continue to show a failure-free survival benefit.^4^, ^5^, ^6^ Guidelines from the American Urologic Association, American Society for Radiation Oncology, and Society of Urologic Oncology now recommend informing men that favorable intermediate-risk prostate cancer can be treated with radiation therapy alone.^7^

The results of this study show decreasing use of ADT from 2004 to 2009 and increasing use of ADT from 2009 to 2016 for men with intermediate-risk prostate cancer who received radiation therapy. The observed increase in ADT use from 2009 to 2016 was primarily composed of an 8.1% absolute increase in ADT use among men with unfavorable intermediate-risk prostate cancer. In comparison, ADT use among patients with favorable intermediate-risk prostate cancer increased modestly by 1.8%. We identified multiple demographic and socioeconomic factors associated with ADT use. Notably, Black men were more likely to receive ADT than were White men, perhaps owing to concerns for increased disease aggressiveness or decreased survival in the Black population.^8^ This is consistent with findings demonstrating that Black men may be less likely to receive de-escalated treatment for prostate cancer, such as active surveillance for low-risk disease.^9^

The pattern of ADT use with respect to age is noteworthy. The use of ADT was associated with older ages compared with men younger than 60 years, who may derive the most benefit. There was an 11%, 24%, and 11% higher likelihood that men aged 60 to <70 years, 70 to <80 years, and 80 to 90 years, respectively, would receive ADT. Although our analysis excluded men who refused or had contraindications to ADT, they may reflect uncaptured patient preferences regarding the adverse effects of ADT (decreased libido, hot flashes, weight gain, cardiovascular risks, etc), notwithstanding its known survival benefits.

Most notably, men treated with BT had a marked decline in ADT use compared with men treated with EBRT. Men treated with EBRT + BT had a 5.3% absolute reduction in ADT use, in contrast to men treated with BT alone, who demonstrated a 23.5% absolute decrease in ADT use between 2004 and 2016. Multiple studies including the ASCENDE-RT randomized trial have demonstrated improved biochemical-free survival with dose-escalated therapy with BT in combination with EBRT.^10^, ^11^, ^12^, ^13^ Similarly, current evidence has demonstrated no benefit of adding ADT to BT alone for men with low-risk and favorable intermediate-risk prostate cancer.^14^, ^15^, ^16^ The results of this study support the rapid adoption of ADT de-escalation recommendations for men receiving BT alone. However, this decrease in ADT use was also demonstrated among patients with unfavorable intermediate-risk prostate cancer receiving BT alone, in contrast to patients receiving EBRT or EBRT + BT. These findings raise concern about potential undertreatment of the subset of patients with unfavorable intermediate-risk disease who receive BT alone. Furthermore, a recent systematic review of predominantly intermediate- and high-risk localized prostate cancer suggests that patients receiving EBRT + BT without ADT may have inferior overall survival compared with those receiving EBRT alone in combination with ADT.^17^

It is unclear why overall ADT use decreased from 2004 to 2009, although this may reflect multifactorial concerns regarding the morbidity of associated adverse effects, effects on quality of life, risk of cardiac toxic effects, or unclear therapeutic benefit.^18^, ^19^, ^20^, ^21^ The increased incorporation of ADT with definitive radiation therapy may reflect clinical adoption after seminal trials demonstrating overall survival benefit with short-term ADT before dose escalated radiation therapy. These trials include post hoc analysis of the RTOG 94 to 08 trial, which demonstrated improvement in overall survival with 4 months of ADT, with benefit predominantly in men with intermediate-risk prostate cancer.^22^ Similarly, long-term data from the Dana Farber Cancer Institute (DFCI) 95096 trial demonstrated an 8-year overall survival improvement of 13%, and the Trans Tasman Radiation Oncology Group 9601 trial demonstrated a 10-year all-cause mortality benefit of 13.2% with 6 months of ADT compared with radiation therapy alone.^23^^,^^24^ However, both of these trials included men with intermediate- and high-risk prostate cancer. Additionally, concerns regarding cardiotoxicity of ADT may have been mitigated by subsequent post hoc analyses of randomized ADT trials demonstrating no association with ADT and cardiovascular risk.^25^^,^^26^

The results of our analysis suggest that nearly 30% of men receiving radiation therapy for favorable intermediate-risk prostate cancer may be overtreated with ADT, and thus, this study has important implications for clinical practice. Conversely, 45% of men with unfavorable intermediate-risk prostate cancer do not receive ADT, which may affect survival. It is unclear why ADT use in this cohort is low, particularly given no significant differences in comorbidity index between men who receive ADT compared with those who do not. This may instead reflect uncaptured patient or physician preferences, rather than clinicopathologic features.

### Limitations

The findings of this study are subject to the inherent biases of its retrospective nature. These data were derived from the NCDB registry, which comprehensively tabulates an estimated 70% of all new cancer diagnoses, thus limiting the scope of our study to approximately two-thirds of men diagnosed with intermediate-risk prostate cancer from 2004 to 2016. Despite the thorough data collected by the NCDB, it does not collect data on imaging that may influence clinical staging, biochemical progression-free survival, metastasis-free survival, prostate cancer–specific mortality, information on ADT duration, baseline genitourinary symptoms, compliance, or adverse effects of treatment. However, because the present study was not intended to report on survival or efficacy, this information would have added significant depth to the analyses but was not vital to our findings.

## Conclusions

The findings of this study highlight that ADT use is variable in men undergoing radiation therapy for intermediate-risk prostate cancer, with data suggesting that several clinical and socioeconomic disparities influence its use. Ongoing review of practice patterns will be needed to assess adoption of clinical guidelines for ADT in intermediate-risk prostate cancer.

## Appendix Supplementary materials

Image, application 1

Image, application 2
