# Supplementary figures and images for: Trends in Androgen Deprivation Use in Men With Intermediate-Risk Prostate Cancer Who Underwent Radiation Therapy
Source: Adv Radiat Oncol. 2022 Feb 4;7(4):100904. doi: 10.1016/j.adro.2022.100904 (PMC9260097; doi:10.1016/j.adro.2022.100904)

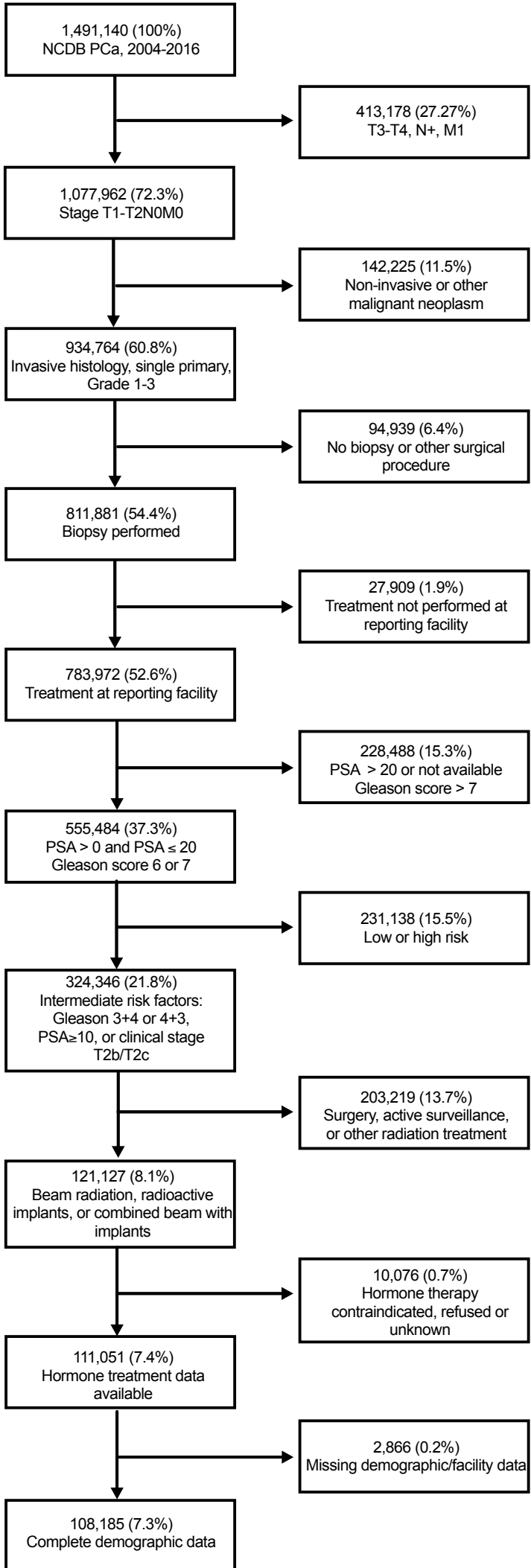

Supplement: Supplementary file 1 [file mmc1.pdf]
