# Supplementary material for: Trends in Androgen Deprivation Use in Men With Intermediate-Risk Prostate Cancer Who Underwent Radiation Therapy
Source: Adv Radiat Oncol. 2022 Feb 4;7(4):100904. doi: 10.1016/j.adro.2022.100904 (PMC9260097; doi:10.1016/j.adro.2022.100904)
Supplement: Supplementary file 2 [file mmc2.docx]

Supplemental Table 1: Multivariable Logistic Regression Model Estimating Androgen Deprivation Therapy Use Among 108,105 Men

|  |  | P Value | |
| --- | --- | --- | --- |
| Variable | Odds Radio (95% CI) | Factor Level | Overall |
| Age |  |  |  |
| <60 | 1 [Reference] |  | < 0.0001 |
| 60 - <70 | 1.11 (1.07 – 1.16) | < 0.0001 |  |
| 70 - <80 | 1.24 (1.18 – 1.29) | < 0.0001 |  |
| 80 - 90 | 1.11 (1.04 – 1.19) | = 0.003 |  |
| Race |  |  |  |
| White | 1 [Reference] |  | < 0.0001 |
| Black | 1.11 (1.07 –1.15) | < 0.0001 |  |
| Other | 1.10 (1.03 –1.17) | 0.007 |  |
| Year of Diagnosis |  |  |  |
| 2004 | 1 [Reference] |  | < 0.0001 |
| 2005 | 0.90 (0.84 - 0.96) | = 0.0008 |  |
| 2006 | 0.85 (0.80 –0.90) | < 0.0001 |  |
| 2007 | 0.74 (0.70 – 0.79) | < 0.0001 |  |
| 2008 | 0.63 (0.59 – 0.68) | < 0.0001 |  |
| 2009 | 0.50 (0.47 – 0.54) | < 0.0001 |  |
| 2010 | 0.54 (0.50 – 0.57) | < 0.0001 |  |
| 2011 | 0.53 (0.50 – 0.57) | < 0.0001 |  |
| 2012 | 0.57 (0.53 – 0.61) | < 0.0001 |  |
| 2013 | 0.52 (0.49 – 0.56) | < 0.0001 |  |
| 2014 | 0.53 (0.50 – 0.57) | < 0.0001 |  |
| 2015 | 0.57 (0.54 – 0.61) | < 0.0001 |  |
| 2016 | 0.60 (0.57 – 0.64) | < 0.0001 |  |
| Gleason Score |  |  |  |
| 3 + 3 | 1 [Reference] |  | < 0.0001 |
| 3 + 4 | 2.20 (2.08 – 2.32) | < 0.0001 |  |
| 4 + 3 | 3.34 (3.09 – 3.66) | < 0.0001 |  |
| Clinical T Stage |  |  |  |
| T1a - T2a | 1 [Reference] |  | < 0.0001 |
| T2b | 1.66 (1.58 – 1.76) | < 0.0001 |  |
| T2c | 1.71 (1.62 – 1.81) | < 0.0001 |  |
| PSA (ng/ml) |  |  |  |
| ≤ 4 | 1 [Reference] |  | < 0.0001 |
| 4 – ≤ 10 | 1.05 (1.01 – 1.09) |  |  |
| 10 – ≤ 20 | 2.13 (2.01 - 2.25) |  |  |
| Primary Payer |  |  |  |
| Private | 1 [Reference] |  | < 0.0001 |
| Medicare/Medicaid/Other Government | 1.13 (1.10 – 1.17) | < 0.0001 |  |
| Uninsured | 1.11 (0.98 – 1.24) | = 0.09 |  |
| Facility Type |  |  |  |
| Academic/Research | 1 [Reference] |  | < 0.0001 |
| Community | 1.64 (1.56 –1.72) | < 0.0001 |  |
| Comprehensive | 1.34 (1.30 – 1.38) | < 0.0001 |  |
| Integrated Network | 1.19 (1.14 – 1.24) | < 0.0001 |  |
| Location Type |  |  |  |
| Metropolitan | 1 [Reference] |  | < 0.001 |
| Urban | 1.04 (1.00 –1.09) | = 0.04 |  |
| Rural | 1.23 (1.12 –1.34) | < 0.001 |  |
| Distance from Facility |  |  |  |
| <=60 miles | 1 [Reference] |  | < 0.0001 |
| 60–120 miles | 0.85 (0.79 –0.92) | = 0.02 |  |
| >120 miles | 0.63 (0.59 –0.68) | < 0.0001 |  |
| Facility Location |  |  |  |
| New England | 1 [Reference] |  | < 0.0001 |
| Central: East North | 0.69 (0.65 –0.73) | < 0.0001 |  |
| Central: East South | 0.58 (0.54 –0.62) | < 0.0001 |  |
| Central: West North | 0.86 (0.80 –0.92) | < 0.0001 |  |
| Central: West South | 0.67 (0.62 –0.73) | < 0.0001 |  |
| Mid Atlantic | 0.81 (0.77 –0.86) | < 0.0001 |  |
| Mountain | 0.64 (0.59 –0.70) | < 0.0001 |  |
| Pacific | 0.58 (0.54 –0.62) | < 0.0001 |  |
| South Atlantic | 0.59 (0.56 –0.62) | < 0.0001 |  |
| Education Level (No High School %) |  |  |  |
| <7% | 1 [Reference] |  | < 0.0001 |
| 7-12.9% | 1.07 (1.03 – 1.11) | 0.0003 |  |
| 13-20.9% | 1.06 (1.02 – 1.11) | 0.0006 |  |
| >=21% | 1.12 (1.07 – 1.18) | <0.0001 |  |
